# Suppression of Th1 and Th17 Responses and Induction of Treg Responses by IL-18-Expressing Plasmid Gene Combined with IL-4 on Collagen-Induced Arthritis

**DOI:** 10.1155/2018/5164715

**Published:** 2018-05-08

**Authors:** Qiaomei Dai, Yang Li, Haiyue Yu, Xiaoyan Wang

**Affiliations:** ^1^Department of Rheumatology and Immunology, The Second Affiliated Hospital of Harbin Medical University, Harbin, China; ^2^Department of Pathology, Heilongjiang University of Chinese Medicine, Harbin, China; ^3^Department of Rheumatology, Qiqihar First Hospital, Qiqihar, China

## Abstract

**Objectives:**

IL-18 is a proinflammatory cytokine with multiple immunoregulatory properties. We studied the effect of IL-18 gene therapy on the development of murine collagen-induced arthritis (CIA).

**Methods:**

Plasmid pCAGGS-IL-18 along or in combination with IL-10 or IL-4 was administered to CIA mice. The incidence and severity of arthritis of the paws were determined by a visual scale. Joint destruction was determined by histology. The levels of a panel of cytokines and transcription factors in the synovium were determined by reverse transcription polymerase chain reaction and quantitative RT-PCR. Quantitative RT-PCR was employed to detect the mRNA expression of TLRs and their pathway on the surface of DCs.

**Results:**

IL-18 gene therapy had no therapeutic effect on CIA mice. Additional coadministration with low dosage of recombinant IL-4 ameliorated the disease progression. Histopathological examination of the joints showed intact cartilage surface in IL-18 gene combined with IL-4-treated mice. The synovium of IL-18 gene combined with rIL4-treated mice had lower expression of TNF-*α*, IFN-*γ*, and IL-17 and higher expression of IL-10. The mechanism of this response appeared to involve modulation of transcription factors FoxP3 and GATA-3. The DCs in the spleen and lymph nodes of IL-18 gene combined with rIL4-treated mice had lower expression of TLR2, MyD88, and NF-kB.

**Conclusions:**

Our findings indicate that pIL-18 gene combined with IL-4 ameliorates arthritis in the CIA mouse by suppression of Th1 and Th17 cytokines and increasing expression of FoxP3 and GATA-3. The plasmid backbone and multiple immunoregulatory properties of IL-18 appear to play a major role in the pIL-18 coadministration with rIL-4-mediated immunomodulation of arthritis through blocking the TLR2/MyD88/NF-kappa B signaling pathway.

## 1. Introduction

Rheumatoid arthritis (RA) is an autoimmune disease characterized by chronic inflammation that results in cartilage damage and bone destruction [1]. The roles of T cells in the pathogenesis of RA are not fully understood. However, previous studies reported that Th1 cytokines (interferon-*γ* [IFN-*γ*], tumor necrosis factor-*α* [TNF-*α*], and interleukin-1 [IL-1]), Th2 cytokines (IL-4 and IL-10), Th17 cytokines (IL-17 and IL-22), and CD4^+^CD25^+^FoxP3^+^ regulatory T cells (Tregs) affect each other and together with cell surface molecules activate synovial macrophages, fibroblast-like cells, and osteoclasts, leading to chronic inflammation and joint destruction [2, 3]. Dendritic cells are professional antigen presenting cells (APCs) that can initiate and regulate T-cell responses. Toll-like receptor (TLR) as the surface molecule of DCs activates innate immunity and plays an important role in RA [4]. TGF-*β* alone induces the Treg transcription factor Foxp3 and is essential for the development of Tregs in the periphery [5]. However, the presence of proinflammatory cytokines such as IL-6, which is induced during infection, inflammation, and injury, inhibits the induction of Foxp3^+^ Tregs and simultaneously promotes Th17-cell differentiation [5, 6].

IL-18 is a proinflammatory cytokine with multiple immunoregulatory properties. IL-18 belongs to the IL-1 ligand superfamily and is structurally and functionally similar to IL-1. IL-18 was originally described as an inducer of Th1 responses [7]. In addition, IL-18 can also stimulate Th2 development and in concert with IL-23 can activate Th17 cells [7, 8]. Other research indicates that IL-18 plays a prominent role in the onset and maintenance of the inflammatory response during RA [9]. Local neutralisation of IL-18 by the naturally occurring IL-18-binding protein (IL-18BP) or systemic neutralisation by specific antibodies ameliorates collagen-induced arthritis (CIA), reduces inflammation, and reduces cartilage erosion [10, 11]. Yao et al. reported that a recombinant virus expressing the IL-18BP/IL-4 fusion protein (AD-IL-18BP/IL-4) suppresses the production and expression of inflammatory cytokines in lipopolysaccharide- (LPS-) stimulated synovial fibroblasts [12]. Another study demonstrated that soluble IL-18 receptor *β* inhibited IL-18 during experimental arthritis, had a major effect on T-cell cytokine balances, and led to aggravation of CIA [9]. Our previous research suggested that recombinant murine IL-18 (rmIL-18) treatment alone ameliorated the progression of arthritis in mice with CIA and that coadministration with low-dose rIL-10 reversed this effect [2].

In this study, we used the murine CIA model to examine the effect of gene transfer of an IL-18-expressing plasmid on suppression of CIA and the possible roles of Treg, Th2, Th1, Th17, and TLRs in the pathogenesis of arthritis.

## 2. Materials and Methods

### 2.1. Reagents

LPS (*Escherichia coli* 0111:84), Bovine type II collagen, and Freund's complete adjuvant (FCA) were purchased from Sigma (St. Louis, MO); RNA PCR Kit (AMV, Ver.3.0 and EX TAQ R-PCR Version 2.1) was from TaKaRa Biotechnology (Dalian, China); Trizol was from Invitrogen Corporation (San Diego, CA); primers and probes were from Pharmacia Biotech (Roosendaal, Netherlands); and murine recombinant IL-10 and IL-4 were from Prospec-Tany Technogene (Rehovot, Israel). CD11c MicroBeads were from Miltenyi Biotec Inc (Bergisch Gladbach, Germany).

### 2.2. Induction of Collagen-Induced Arthritis (CIA)

Male DBA/1 mice (8–12 weeks old, purchased from Slac Laboratories, Shanghai, China) were housed in a specific pathogen-free environment with a 12 h light-dark cycle. For the CIA model, 100 *μ*g of bovine type II collagen dissolved in 0.05 M acetic acid was emulsified with an equal volume of FCA and administered intradermally at the base of the tail. On day 21, the mice were boosted by intraperitoneal injection of 100 *μ*g of bovine type II collagen dissolved in phosphate buffered saline (PBS). On day 28, forty mice with no macroscopic signs were treated by an additional intraperitoneal injection of LPS (40 *μ*g). Forty mice were sacrificed on day 60 for analysis. This study was reviewed and approved by the Ethics Committee for Experimental Animals of Harbin Medical University, and all animals were treated according to the guidelines of the animal ethical committee.

### 2.3. Assessment of Arthritis

The onset of arthritis was considered to be the day that erythema and/or swelling were first observed. Mice were considered to have arthritis when significant changes in redness and or swelling were noted in the digits or in other parts of at least 2 paws. At later time points, ankylosis was also included in the macroscopic scoring. Cumulative scoring depending on redness, swelling, and in later stadium ankylosis was as follows: 0—no changes; 0.25—1 to 2 toes red or swollen; 0.5—3 to 5 toes red or swollen; 0.5—swollen ankle; 0.5—swollen footpad; 0.5—severe swelling and ankylosis. The macroscopic score was assessed by two independent, blinded observers [10].

### 2.4. Histology

After animal sacrifice, whole knee joints were removed and specimens were fixed for 4 days in 4% formalin, decalcified in 5% formic acid, and processed for paraffin embedding. Tissue sections were stained with hematoxylin and eosin (H&E) or Masson stain. Histopathology was used to assess the occurrence of cartilage destruction and bone erosion.

### 2.5. Gene Therapy

After animal sacrifice, plasmid DNA expressing murine IL-18 was prepared as previously described [13]. On day 28, forty mice without signs of arthritis were given intramuscular injections of pIL-18 (10 *μ*g), pIL-18 (10 *μ*g) combined with IL-4 (0.1 *μ*g), pIL-18 (10 *μ*g) combined with IL-10 (0.1 *μ*g), or PBS as control. A booster injection of the same dosage was given two weeks later. The same volume of hyaluronidase IV (Sigma, St. Louis, MO) was injected 10 min before these injections to improve the plasmid expression. In a repeat experiment, fifty mice without signs of arthritis were given intramuscular injections of pIL-18 (10 *μ*g) combined with IL-4 (0.1 *μ*g), IL-4 (0.1 *μ*g), plasmid (pCAGGS) combined with IL-4 (0.1 *μ*g), or with plasmid (pCAGGS) and PBS as control.

### 2.6. Isolation of Spleen and Lymph Nodes CD11c+ DCs

Mice were sacrificed by cervical dislocation. The spleens and popliteal lymph nodes were removed, any fatty tissue was trimmed away, and spleens were washed three times with PBS. The spleens and popliteal lymph nodes were laid on a 200-mesh pore size cell strainer, and using the barrel from a 1 mL syringe the spleens and lymph nodes were pressed through the strainer until only a small amount of fibrous tissue remained in the strainer. CD11c+ cells were isolated with the CD11c MicroBeads according to the manufacturer's instructions [14].

### 2.7. RNA Preparation, Reverse Transcription Polymerase Chain Reaction, and Quantitative RT-PCR

The patella and adjacent synovium were dissected immediately after animal sacrifice. The synovium samples were immediately frozen in liquid nitrogen and ground into powder with a tissue grinder, and total RNA was extracted in 1 mL Trizol reagent, similar to the procedure used for cartilage samples. The levels of a panel of cytokines and transcription factors in the synovium were determined by reverse transcription polymerase chain reaction and quantitative RT-PCR. The CD11c+ cell suspension was counted and RNA was extracted. The expression of TLRs and their pathway on the surface of DCs was determined by quantitative RT-PCR. Primer sequences and conditions for PCR were from the paper described previously [11]. Primer sequences for PCR were as follows: *β*-actin, forward: 5′-AGC GGT TCC GAT GCC CT-3′; reverse: 5′-AGA GGT CTT TAC GGA TGT CAA CG-3′; T-bet, forward: 5′-GCC AGG AAC CGC TTA TAT G-3′; reverse: 5′-TTG TTG GAA GCC CCC TTG-3′; GATA-3, forward: 5′-AGG TGG ACG TAC TTT TTA ACA TCG; reverse: 5′-GCT AGC CCT GAC GGA GTT TTC-3′; IFN-*γ*, forward: 5′-TGA ACG CTA CAC ACT GCA TCT TGG; reverse: 5′-CGA CTC CTT TTC CGC TTC CTG AG-3′; Foxp3, forward: 5′-GGC CCT TCT CCA GGA CAG A; reverse: 5′-GCT GAT CAT GGC TGG GTT GT-3′; interleukin-17A, forward: 5′-AGT GAA GGC AGC AGC GAT CAT-3′; reverse: 5′-CGC CAA GGG AGT TAA AG-3′; TNF-*α*, forward: 5′-TCT CAT CAG TTC TAT GGC CC-3′; reverse: 5′-GGG AGT AGA CAA GGT ACA AC-3′; IL-18, forward: 5′-ACC GAA TTC ACT GTA CAA CCG CAG TAA TAC GGA 3′; reverse: 5′ GCC TCT AGA GTG AAC ATT ACA GAT TTA TCC CCA 3′; IL-10, forward: 5′GAA GAC CCT CAG GAT GCG 3′; reverse: 5′ CCA AGG AGT TGT TTC CGT TA 3′; TLR-2, forward: 5′-GCA AAC GCT GTT CTG CTC AG-3′; reverse: 5′-AGG CGT CTC CCT CTA TTG TAT T-3′; TLR-4, forward: 5′-ATG GCA TGG CTT ACA CCA CC-3′; reverse: 5′-GAG GCC AAT TTT GTC TCC ACA-3′; TLR-9, forward: 5′-ATG GTT CTC CGT CGA AGG ACT-3′; reverse: 5′-GAG GCT TCA GCT CAC AGG G-3′; MyD88, forward: 5′ TCA TGT TCT CCA TAC CCT TGGT-3′; reverse: 5′-AAA CTG CGA GTG GGG TCA G-3′; TRIF, forward: 5′-TGT CTG TCA GGA GGT GCT CAA-3′; reverse: 5′-CGT TCC GGA CAT GCT CTT TC-3′; NF-*κ*B, forward: 5′-GGA GGC ATG TTC GGT AGT GG-3′; reverse: 5′-CCC TGC GTT GGA TTT CGT G-3′. Transcripts were quantified using the EX TAQ R-PCR. The PCR was initiated for 2 min at 95°C, continued with 40 cycles of 10 sec at 95°C and 40 sec at 60°C. The fold change in expression of each gene was calculated using the ΔΔCt method, with the housekeeping gene *β*-actin mRNA as an internal control.

### 2.8. Statistical Analysis

Data are presented as the means ± SDs. The statistical significance of differences was analyzed by Student's *t*-test and Wilcoxon rank test using SPSS software 13.0 for Windows (SPSS, USA). A *p* value less than 0.05 was considered significant.

## 3. Results

### 3.1. IL-18 Gene Combined with IL-4 Inhibits CIA by Skewing the Balance of Th1/Th2/Th17 to a Th2 Type

**Figure 1:**
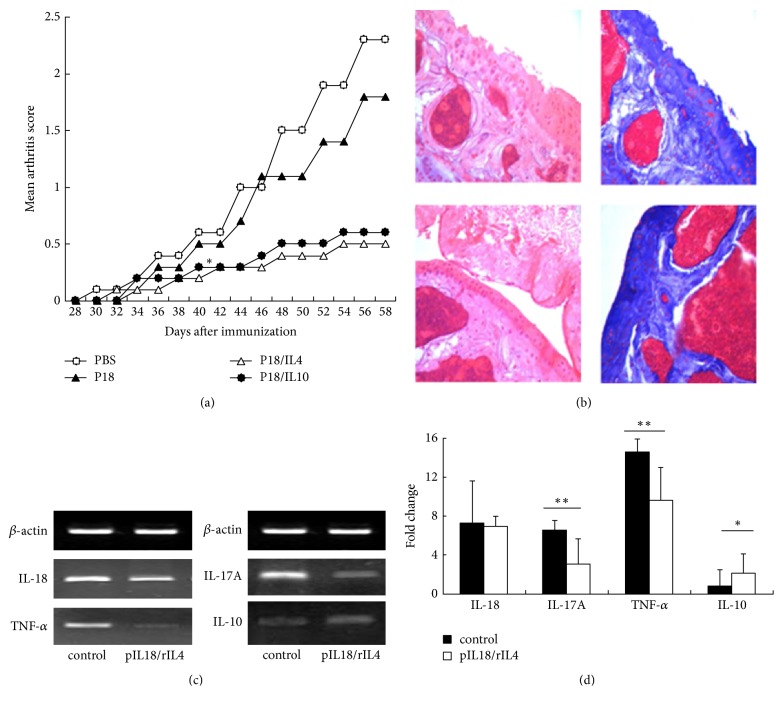
*Effect of pIL-18 combined with rIL4 on CIA mice*. Male DBA/1 mice were intradermally immunized with type II collagen emulsified in an equal volume of Freund's complete adjuvant (FCA) and given intraperitoneal booster injections with type II collagen in PBS after 21 days. At day 28, mice were given intramuscular injections of 10 *μ*g of pIL-18 (alone or in combination with rIL4 or rIL10) or PBS as control, with a booster at the same dosage 14 days later. (a) Mean arthritis macroscopic scores of mice treated with pIL-18 + rIL4 and pIL-18 + rIL10 were significantly lower than the control from day 44 onwards (^*∗*^*p* < 0.05 versus control by Wilcoxon rank test). Each point represents the mean ± SD of ten mice. (b) Representative histological findings (left: H&E staining ×40; right: Masson staining ×40). Mice were sacrificed on day 60, and ankle and knee joints were removed. The upper two images show severe cartilage surface disruption in CIA mice given the PBS control; the lower two images show intact cartilage surface in CIA mice given pIL-18 combined with rIL4. (c) Representative reverse transcription PCR results showing the expression of IL-18, TNF-*α*, IL-17A, and IL-10 in CIA mice given the control PBS or pIL-18 combined with rIL4 (60 days after immunization). (d) Changes in the expression of TNF-*α*, IL-18, IL-10, and IL-17A were determined by real-time PCR. ^*∗*^*p* < 0.05 versus control by Student's *t*-test; ^*∗∗*^*p* < 0.01 versus control by Student's *t*-test.

Previous studies demonstrated that recombinant IL-18 coadministration with low dosage rIL-4 does not prevent the progression of CIA [2]. Thus, we studied the role of IL-18 gene therapy in murine CIA on day 28 after immunization and administered a boost two weeks later (Figure 1). Surprisingly, IL-18 gene therapy combined with IL-4 significantly reduced the mean arthritis score of our murine model (Figure 1(a)). Additional coadministration with low dosage rIL-10 had same effect that accord with our previous studies. Consistent with these results, histopathological examination of the joints indicated that mice given the control PBS had severe disruption of the cartilage surface, but that mice given pIL-18 combined with IL-4 had intact cartilage surface (Figure 1(b)). Next, we measured gene expression in synovial specimens to investigate the possible mechanism of IL-18 gene combined with rIL4 therapy (Figures 1(c) and 1(d)). The synovium of IL-18 gene combined with rIL4-treated mice had significantly lower expression of TNF-*α* and IL-17A and significantly higher expression of IL-10 (Figures 1(c) and 1(d)).

### 3.2. The Plasmid Backbone Contributes to the Therapeutic Effect of IL-18 Gene Combined with rIL-4

**Figure 2:**
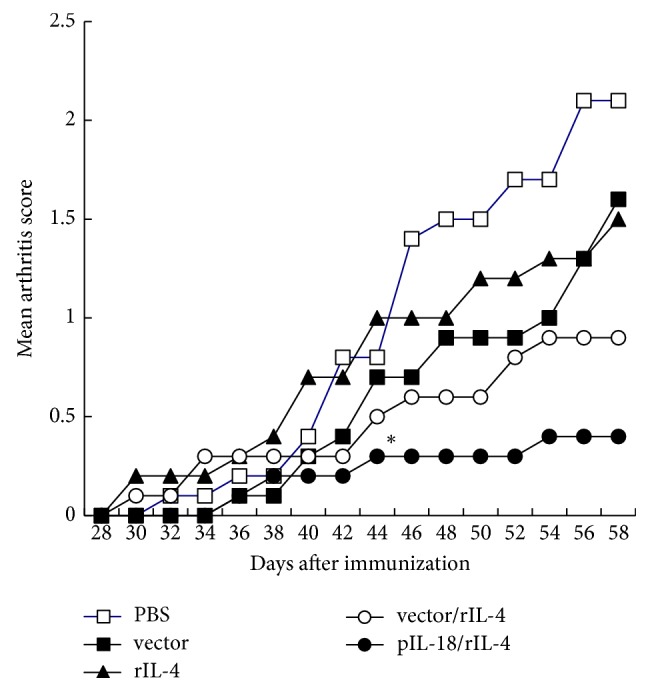
*Effect of various vectors on the mean arthritis macroscopic score of CIA mice*. DBA/1 mice were immunized with type II collagen on day 0 and day 21. On day 28, mice were treated with PBS, 10 *μ*g of control vector, 10 *μ*g of vector + 0.1 *μ*g rIL-4, 0.1 *μ*g of rIL-4, or 10 *μ*g of pIL-18 + 0.1 *μ*g rIL-4. A booster treatment was given with the same dosage after two weeks. Each point represents the mean ± SD of ten mice. Mean arthritis macroscopic scores of mice treated with pIL-18 + rIL4 were significantly lower than the control from day 44 onwards. ^*∗*^*p* < 0.05 versus control by Wilcoxon rank test.

Previous research has shown that treatment with rIL-18 and rIL-4 had no therapeutic effects on murine CIA model [2]. The present study, however, showed that treatment with pIL-18 combined with rIL-4 was markedly protective. We hypothesized that this difference was due to the plasmid vector in pIL-18. Thus, we injected rIL-4 into CIA mice with or without an empty plasmid vector (Figure 2). The results indicate significant exacerbation of arthritis in mice treated with the control vector or rIL-4 but that coadministration of the control vector and rIL-4 had lower macroscopic arthritis score than the other three groups except pIL-18 combined with IL-4 group.

### 3.3. Interaction of the P IL-18 with rIL-4

**Figure 3:**
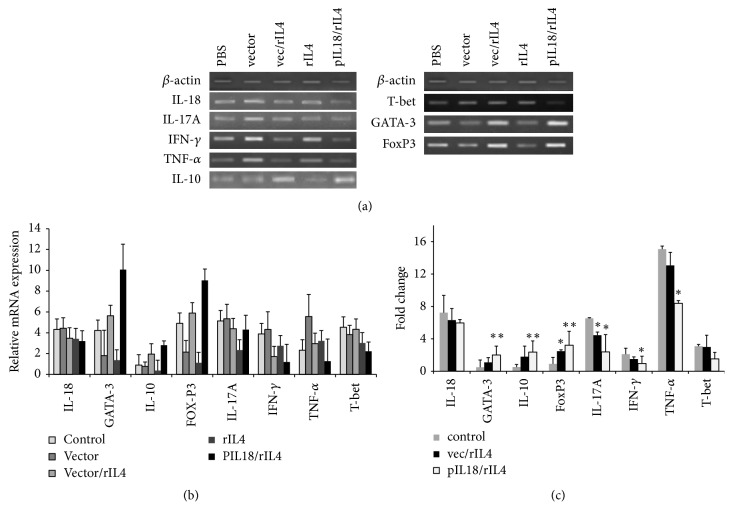
*Effect of various vectors on cytokine expression in the synovia of CIA mice*. (a) Representative results showing changes in the expression of IL-17A, IL-18, IFN-*γ*, TNF-*α*, IL-10, FoxP3, T-bet, and GATA-3, determined by reverse transcription PCR analysis at 60 days after immunization. The lower part displays the density histogram data from three separated RT-PCR analyses (mean ± SE), which represents the relative expression of T-bet, GATA-3, TNF-*α*, IL-17A, IL-18, IFN-*γ*, IL-10, and FoxP3. (b) Changes in the expression of T-bet, GATA-3, TNF-*α*, IL-17A, IL-18, IFN-*γ*, IL-10, and FoxP3 were determined by real-time PCR. ^*∗*^*p* < 0.05 versus control by Student's *t*-test; ^*∗∗*^*p* < 0.01 versus control by Student's *t*-test.

CIA mice treated with rIL-4 alone or plasmid vector alone had lower levels of IL-10 and higher levels of IFN-*γ* and TNF-*α* than mice given empty plasmid + rIL-4 or pIL-18 with rIL-4 (Figures 3(a) and 3(b)). Disruption of the balance of Th1, Th2, and Th17 cells is associated with progression of RA [5]. Thus, we investigated the expression of different master regulators of these T-cell immune responses. The results indicated that pIL-18 and rIL-4 therapy dramatically increased the expression of GATA-3 and FoxP3 and decreased the expression of IL-17A (Figures 3(a) and 3(b)).

### 3.4. Expression of TLRs and Their Pathways on DCs of Spleen and Lymph Nodes

**Figure 4:**
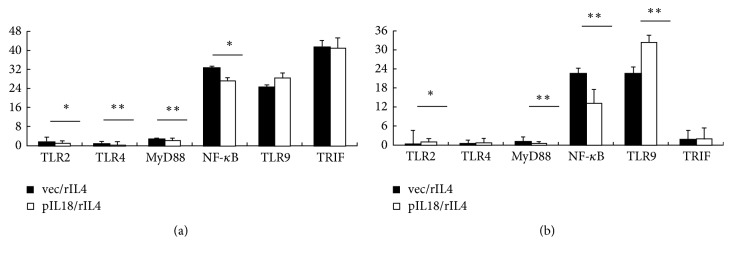
*Effect of various vectors on expression of TLRs and their pathways on DCs of spleen and lymph nodes*. (a) Representative results showing changes in the expression of TLR2, TLR4, MyD88, NF-*κ*B, TLR9, and TRIF in spleen DCs, determined by real-time PCR analysis at 60 days after immunization. (b) Changes in the expression of TLR2, TLR9, MyD88, NF-*κ*B, TLR4, and TRIF in lymph node DCs were determined by real-time PCR. ^*∗*^*p* < 0.05 versus control by Student's *t*-test; ^*∗∗*^*p* < 0.01 versus control by Student's *t*-test.

Finally, we investigated the mechanism of the interaction of the PIL-18 and rIL-4 (Figure 4). The expression levels of TLR2, TLR4, MyD88, and NF-*κ*B in the spleen DCs of PIL-18 and rIL-4 group were significantly lower than those in the spleen DCs of vector + rIL-4 group; however there was no statistical difference in the expressions of TLR9 and TRIF (Figure 4(a)). The expressions of TLR2, TLR9, MyD88, and NF-*κ*B in the lymph nodes DCs of PIL-18 and rIL-4 group were significantly lower than those in the lymph nodes DCs of vector + rIL-4 group, while TLR4 and TRIF were not significantly different (Figure 4(b)).

## 4. Discussion

Various animal models of autoimmune diseases have demonstrated the importance of IL-18 as an immune regulatory cytokine [15]. A recent study reported that treatment of experimental autoimmune encephalomyelitis (EAE) mice with the eukaryotic plasmid DT390-IL-18-Sra (encoding recombinant immunotoxin DT390-IL-18) suppressed clinical and histopathological progression of disease [16]. Our previous report showed that combined treatment of low-dose IL-18 with IL-10 can prevent the progression of arthritis in CIA mice and that this is mediated by a GATA-3-dependent mechanism [2]. In the present study, we demonstrated for the first time that IL-18-expressing plasmid gene combined with IL-4 skewed the balance of Th1/Th2/Th17 to a Th2 type and increased GATA-3 and Foxp3 expression on collagen-induced arthritis.

Previous research has described IL-18 as an inducer of Th1 responses and a stimulator of Th2 development in different cytokine microenvironments [17]. IL-18 can promote Th1 cell proliferation and IFN-*γ* production with no need for TCR activation, so it can be considered an endogenous activator of Th1 cells [18]. IL-18 can also induce IFN-*γ* production from NK cells and seems to play an early and nonredundant role in priming NK cells for optimal production of IFN-*γ* in response to IL-12 [19]. In the absence of IL-12, IL-18 induces Th2 cytokines from NK and NKT cells and induces innate allergic mediators, such as basophils and in some cases mast cells and eosinophils [19]. IL-18 can also drive Th2 effector cytokine production from activated Th1 cells, and these so-called “super Th1 cells,” which produce IFN-*γ*, IL-9, and IL-13, are implicated in the IL-18-mediated promotion of allergic-like pathology [20]. Other research has demonstrated that IL-18 + IL-4 can reduce the differentiation of Th1 cells, IL-18 + IL-10 induces Th2 responses to secrete Th2 cytokines IL-4 [21], IL-18 + IL-10 + IL-4 can reduce Th1 cell secretion of TNF-*α* and IFN-*γ*, and the inhibition of TNF-*α* promotes the population of Treg cells [22, 23]. In addition, IL-18 in concert with IL-23 can activate Th17 cells [8, 24]. Taken together, this indicates that IL-18 is a special cytokine with multiple immune-regulating potentials.

In this report, the synovium of IL-18 gene combined with rIL4-treated mice had significantly lower expression of TNF-*α*, IL-17A, and IFN-*γ* and significantly higher expression of IL-10. Our previous report showed that IL-18 receptor (IL-18R) *α* expression in patella with adjacent synovium in CIA mouse is downregulated by the combined treatment of rIL-18 with IL-4 [2]. This is expected to contribute to the downregulation of Th1 immune responses. Although rIL-18/IL-4 treatment had a slight suppressive effect on the macroscopic arthritis score and incidence of arthritis in our previous research, it did not reach statistical significance [2]. Moreover, combined treatment of empty plasmid vector and recombinant IL-4 had a slight suppressive effect on the macroscopic arthritis score higher than pIL-18 combined with IL-4 therapy. As we show here, pIL-18 combined with rIL-4 ameliorates arthritis in CIA mice. Thus, the differences between pIL-18 and rIL-18 must be examined.

Previous research has reported that pIL-18 containing CpG motifs can activate signaling of Toll-like receptor 9 (TLR9) [13]. Dendritic cells (DCs) express TLR9 intracellularly and specifically recognize the unmethylated CpG motif to develop Th1 immune responses in* L. major*-infected susceptible BALB/c mice [13, 25]. Thus, TLR9 actuates the expression of inflammatory cytokines via activation of NF-*κ*B. The NF-*κ*B signaling pathway has an important role in the induction and modulation of suppressive function of natural Treg when they are confronted with TLR4-stimulating agents, such as Gram-negative bacteria [26]. A previous study confirmed that human plasmacytoid DCs activated by CpG oligodeoxynucleotides induce the generation of CD4^+^CD25^+^ regulatory T cells [27]. TLR9 signaling may protect against lupus by modulating the activity of regulatory T cells [28, 29].

Treg cells play an active role in preventing the spontaneous development of systemic autoimmunity and previous research has focused on whether deficiencies in Treg cells activity might contribute to the development of autoimmune diseases such as RA [30]. Treg cells are present in the synovial fluid of RA patients and are potent suppressors of responder T-cell proliferation and TNF and IFN-*γ* production; in addition, activated T cells in the synovium also seemed to be suppressed by Treg cells [1, 31]. On the other hand, IL-17 is a key driver of inflammation, and it is present in the rheumatoid synovium of the joints of arthritic mice [32, 33]. Mice deficient in IL-17 have reduced severity of arthritis, and those with increased IL-17 level have exacerbated disease [34, 35]. At the molecular level, Th17 and Treg transcription factors ROR*γ*t/ROR*α* and FoxP3 can bind to each other and inhibit its function [32, 33]. IL-2, a growth factor for Tregs, inhibits the differentiation of Th17-cells, whereas IL-21, which promotes Th17 differentiation, inhibits Treg expansion [36]. Microbial stimuli signaling through TLR2-6 induces Treg to secrete TGF-*β* and IL-10 and to activate dendritic cells (DCs) [21]. TGF-*β* induces the FoxP3 expression but also stable FoxP3 expression [1]. Some evidence indicates that inhibition of TNF in RA promotes the emergence of a Treg cell population that can suppress effector T cells through TGF-*β* and IL-10-dependent pathways [37].

In the present study, we found that IL-18 gene combined with rIL-4 and vector combined with rIL-4 increased the expression of FoxP3. Thus, the plasmid backbone, which contains the CpG motif signaling with TLR9, appears to play a major role in stimulating Treg cells, thereby slowing the progression of arthritis in our CIA model. The present study of a CIA model indicates that pIL-18 coadministration with rIL-4 delivery can activate Treg cells by increasing expression of FoxP3, which presumably leads to the inhibition of Th1 and Th17 inflammatory cytokines.

Although the plasmid backbone had the Treg cells effect, the use of the vector alone or in combination with IL-4 had no therapeutic effect. TNF and IFN-*γ* (Th1 cytokines) production in synovium were found to decrease after the vector alone or in combination with IL-4 treatment, but it did not reach statistical significance, whereas marked decreases in TNF-*α*, IFN-*γ*, and IL-17 expression were detected in PIL-18/rIl-4-treated mice compared with that of control group. Enhanced levels for Foxp3 and IL-10 were more impressive after PIL-18/rIl-4 treatment. IL-18 can promote Th1 cell proliferation. IL-18 receptor (IL-18R) *α* expression in patella with adjacent synovium in CIA mouse is downregulated by the combined treatment of rIL-18 with rIL-4 in our previous research [2]. Th1 response can be inhibited by the effect of rIL-18 + rIL-4 and Treg effect of plasmid backbone in this research. IL-18 gene combined with IL-4 inhibits CIA by skewing the balance of Th1/Th2 to a Th2 type. IL-10 is a Th2 cytokine. The expression of IL-10 in synovium increased which further induced the expression of Treg. Th1 and Th17 responses can be inhibited by Treg cells. The transcription factors GATA-3 and T-bet have been described as “master switches” in the development of Th1 and Th2 cells; expression of T-bet or GATA-3 not only determines Th differentiation, but also can actually override the influence of exogenous polarizing stimuli or previous polarization [38, 39]. A significant increase in GATA-3 expression was demonstrated in PIL-18/rIl-4-treated group compared with that in control group. Increased expression of GATA-3 indicated that IL-18 gene combined with IL-4 inhibits CIA by skewing the balance of Th1/Th2/Th17 to a Th2 type.

To further understand the mechanism of pIL-18 + rIL-4 on CIA, the expression levels of TLRs and their pathway on DCs of the spleen and lymph nodes were detected. The results indicated that pIL-18 and rIL-4 therapy dramatically decreased the expressions of TLR2, TLR4, MyD88, and NF-*κ*B in the DCs of spleen. The expression levels of TLR2, TLR9, MyD88, and NF-*κ*B in the lymph nodes DCs of PIL-18 and rIL-4 were significantly lower than those in vector + rIL-4 group. There were no statistical differences in the expressions of TRIF in the spleen and lymph nodes. The TLRs in DCs activated can cause costimulatory molecules increases and proinflammatory cytokines secretion [40]. The TLRs signaling involves the MyD88 pathway and the TRIF pathway [41]. The results indicated that PIL-18 combined with rIL-4 played a therapeutic role by blocking the TLR2/MyD88/NF-kappa B signaling pathway.

In summary, pIL-18 coadministration with rIL-4 treatment protected CIA mice by significantly reducing the severity of arthritis and the production of proinflammatory cytokines. On the one hand, the effect appeared to be mediated by multiple immune-regulating potentials of IL-18 [2]; on the other hand, the effect appeared to be mediated by modulation of the activity of regulatory T cells. The plasmid backbone and multiple immune-regulating potentials of IL-18 appeared to play the major role in the pIL-18 coadministration with rIL-4-mediated immunomodulation of arthritis, resulting in inhibition of Th17 and Th1 inflammatory immune responses and improving anti-inflammatory mediators by a GATA-3-dependent mechanism. The plasmid backbone and multiple immunoregulatory properties of IL-18 appeared to play the major role in the pIL-18 coadministration with rIL-4-mediated immunomodulation of arthritis through blocking the TLR2/MyD88/NF-kappa B signaling pathway.
